# Single Cell Gene Transcriptome Analysis of Ovarian Mature Teratomas

**DOI:** 10.3389/pore.2021.604228

**Published:** 2021-04-16

**Authors:** Sun Shin, Youn Jin Choi, Seung-Hyun Jung, Yeun-Jun Chung, Sug Hyung Lee

**Affiliations:** ^1^ Department of Integrated Research Center for Genome Polymorphism, The Catholic University of Korea, Seoul, Korea; ^2^ Department of Microbiology, The Catholic University of Korea, Seoul, Korea; ^3^ Department of Cancer Research Center, The Catholic University of Korea, Seoul, Korea; ^4^ Department of Gynecology/Obstetrics, The Catholic University of Korea, Seoul, Korea; ^5^ Department of Biochemistry, The Catholic University of Korea, Seoul, Korea; ^6^ Department of Pathology, The Catholic University of Korea, Seoul, Korea

**Keywords:** teratoma, expression profile, single cell, ovarian tumor, transcriptome

## Abstract

Teratoma is a type of germ cell tumor that originates from totipotential germ cells that are present in gonads, which can differentiate into any of the cell types found in adult tissues. Ovarian teratomas are usually mature cystic teratomas (OMCTs, also known as dermoid cysts). Chromosome studies in OMCTs show that the chromosomes are uniformly homozygous with karyotype of 46, XX, indicating that they may be parthenogenic tumors that arise from a single ovum after thefirst meiotic division. However, the tissues in OMCTs have been known to be morphologically and immunophenotypically identical to the orthotopic tissues. Currently, expression profiles of tissue components in OMCTs are not known. To identify whether OMCT tissues are expressionally similar to or different from the orthotopic tissues, we adopted single-cell RNA-sequencing (scRNA-seq), and analyzed transcriptomes of individual cells in heterogenous tissues of two OMCTs. We found that transcriptome profiles of the OMCTs at single cell level were not significantly different from those of normal cells in orthotopic locations. The present data suggest that parthenogeneticlly altered OMCTs may not alter expression profiles of inrivirual tissue components in OMCTs.

## Introduction

Teratomas are germ cell tumors commonly composed of multiple cell types derived from one or more of the three germ layers and, in many series, the most common excised ovarian tumor [1, 2]. Ovarian teratomas are usually mature cystic teratomas (OMCTs, also known as dermoid cysts), which account for about 11% of all ovarian tumors [1, 2]. In OMCTs, ectodermal derivatives, including *epidermis* and hair follicles are most prominent, but mesodermal and endodermal derivatives are also found mixedly together [1, 2]. These tissues are known to be histologically identical to the orthotopic tissues. OMCTs are diploid, and cytogenetic study demonstrates that they almost always have 46, XX karyotype [3–5]. Also, they are usually homozygous, indicating that they derive most often from a ovum (parthenogenic) that has completed meiosis I but not meiosis II [3–5]. OMCTs harbor distinct methylation profiles of imprinted genes with high and low levels of methylation for maternally and paternally imprinted genes, respectively, supporting the parthenogenic origin [6].

In addition to the parthenogenic alteration, there exists evidence that suggests epigenetic alterations in OMCTs. For example, there is expressional difference in microRNAs between OMCTs and normal ovarian tissues [7]. OMCTs of both mice and human srongly express transcriptional regularor HDAC1, which can be a novel marker for benign teratomas [8]. To our knowledge, transcription profiles of OMCTs have not yet been repprted probably due to the technological limitations with the heterogenous tissue components, which would lead to averaging of expression data. Single-cell RNA-sequencing (scRNA-seq) allows researchers to obtain transcriptome of individual cells, which might further identify population of heterogenous cells by avoiding expression data averaging [9]. In this study, we studied two OMCTs by scRNA-seq that analyzed transcriptomes on a cell-by-cell basis with next-generation sequencing (NGS) cDNA library.

## Materials and Methods

### Tissue Isolation

Ovarian cystectomy specimens were collected from two patients (26 year-old and 48 year-old females) pathologically confirmed as mature cystic teratoma (OMCT). They were separately minced into fragments and digested with collagenase/dispase (Roche Diagnostics, Mannheim, Germany) and DNase I (Roche Diagnostics) for 30 min at 37°C with agitation. The dissociated cell suspension was filtered through 70 μm strainer, washed with phosphate-buffered saline, and centrifuged at x400g for 5 min. The cell pellet was resuspended in RPMI 1640 medium for further use.

### Single-Cell Library Preparation, Sequencing and Pre-processing

Chromium Single Cell 3′ v3 (10x Genomics, Pleasanton, CA) library preparation was performed according to the manufacturer’s protocol. Each library from the two OMCTs was separately sequenced on the Illumina Hiseq platform (Illumina, San Diego, CA) to achieve around 70,000 reads per cell. FASTQ files were processed using Cell Ranger 3.1.0 (10x Genomics) analysis pipeline and were applied to generate a digital gene-cell matrix. Briefly, the files were aligned to the human GRCh38 reference genome followed by unique molecular identifier (UMI) and barcode counting, constructing the UMI count matrices.

### Quality Control and Clustering Analysis

Raw UMI-counts were further analyzed using Seurat R package version 3.1.5 [10]. Briefly, cells with fewer than 200 genes, more than 7,000 genes, or more than 20% mitochondria content were excluded for each sample. Filtered gene-barcode matrices of the two samples were integrated to remove batch effects across different patients using Seurat FindIntegrationAnchors and IntegrateData function [10]. Then uniform manifold approximation and projection (UMAP) was performed on the top 30 principal components for visualizing the cells. Meanwhile, graph-based clustering was performed on the PCA-reduced data for clustering analysis. The resolution was set to 0.9 to obtain a finer result. Finally, the Wilcoxon rank sum test was used to identify differentially expressed genes (DEGs) in each cluster with those in all other clusters using Seurat FindAllMarkers function. Additionally, gene ontology analysis was performed with the top 50 DEGs of each cluster using MSigDB [11] to investigate the functional profiles for genes and gene clusters. We used the SingleR [12] and the Gene Expression Deconvolution Interactive Tool (GEDIT) (http://webtools.mcdb.ucla.edu/) to annotate the clusters and predict the cell type composition.

## Results

**FIGURE 1:**
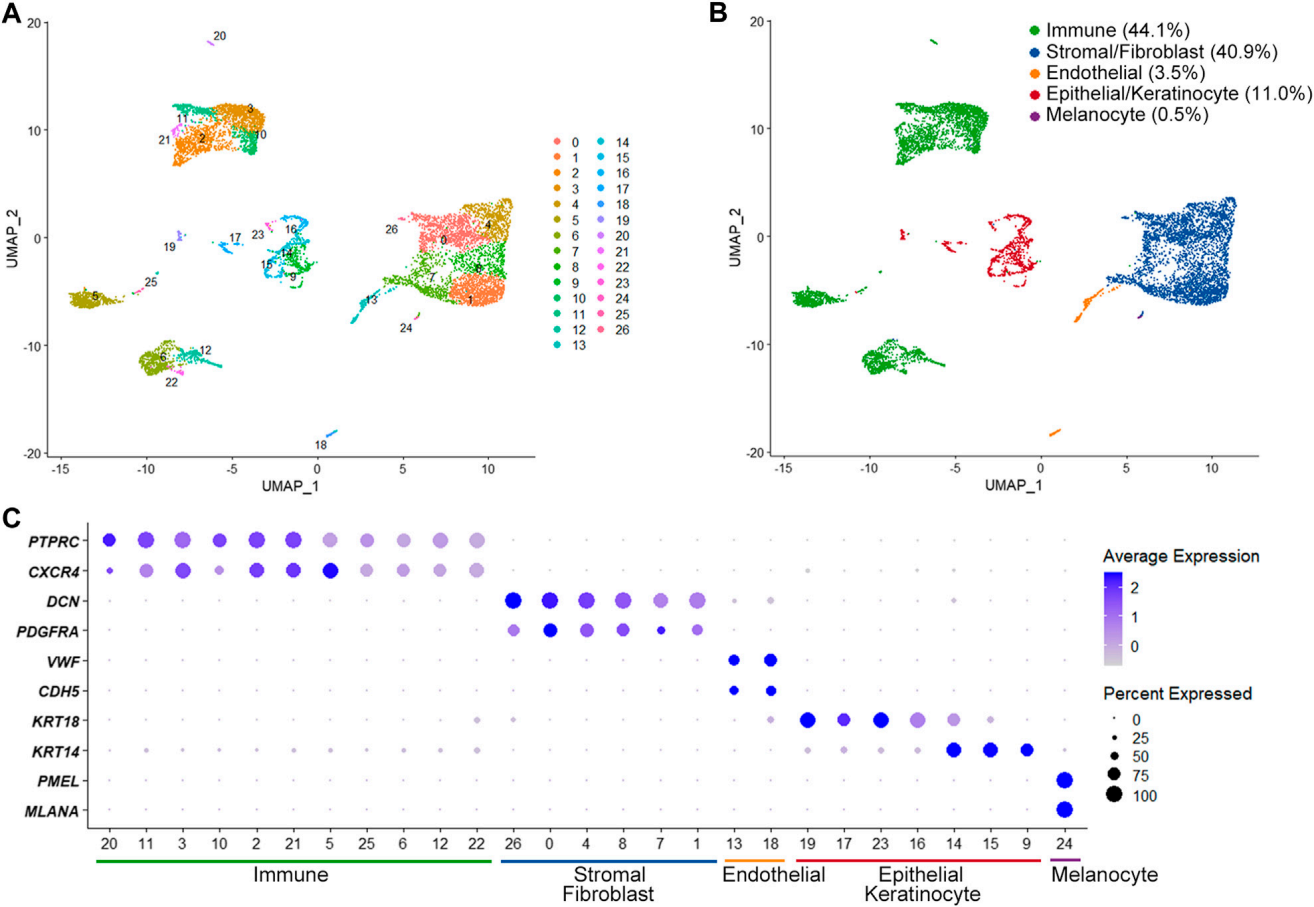
Single-cell transcriptome analysis of ovarian mature cystic teratomas. **(A)** Unsupervized clustering from merged scRNA-seq data of two ovarian mature cystic teratomas. Each dot represents an individual cell, colored according to each cluster. **(B)** Main cell clusters identified with the cell type-specific marker genes. **(C)** Dot plot showing expression of lineage-specific marker genes. The color and size of each dot represents the average gene expression value and the proportion of expressed cells of a given gene in each cluster.

The scRNA-seq identified a total of 21,652 expressed genes in 8,900 cells from two OMCTs (a median of 1,659 genes per cell). We were able to define different cell populations constituting the OMCTs using UMAP and unsupervized clustering (Figure 1A), which showed 27 distinct clusters of immune cells (*PTPRC*, *CXCR4*), stromal cells/fibroblasts (*DCN*, *PDGFRA*), endothelial cells (*VWF*, *CDH5*), epithelial cells/keratinocytes (*KRT18*, *KRT14*) and melanocytes (*PMEL*, *MLANA*) (Figures 1B,C). Immune cells included T cells, B cells and macrophages according to *CD3D*, *IGKC*, and *AIF1* expressions, respectively (Supplementary Figure S1).

**FIGURE 2:**
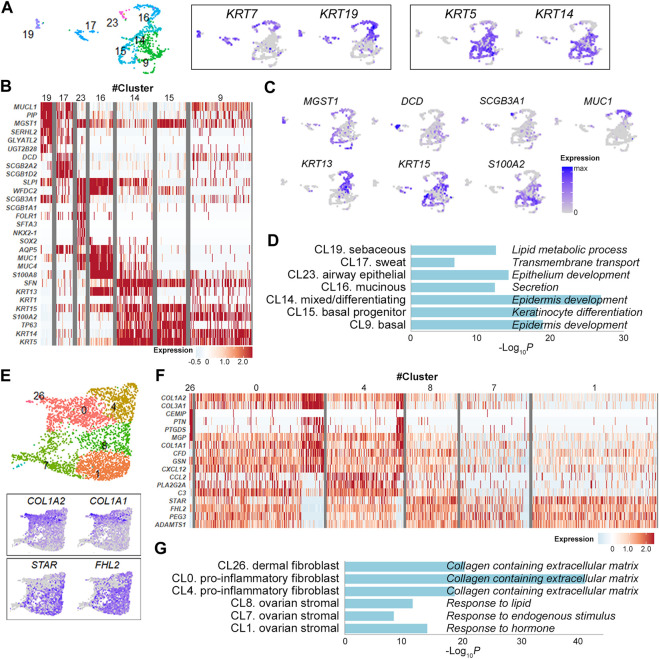
Identification of epithelial cell and fibroblast/stromal cell clusters. **(A)** Epithelial cell clusters with the relative expression of keratin isoform on the UMAP plot. Each dot represents an individual cell. **(B)** Heatmap showing representative marker genes for each epithelial cell cluster. **(C)** UMAP plots showing the relative expression of selected marker genes from each epithelial cell cluster. **(D)** Gene ontology analysis of epithelial cell clusters. The graph shows *p* values for the most significant ontology term for each cluster. **(E)** Fibroblast/stromal cell clusters with the relative expression of selected marker genes on the UMAP plot. **(F)** Heatmap showing representative marker genes for each fibroblast/stromal cell cluster. **(G)** Gene ontology analysis of fibroblast/stromal cell clusters. The graph shows *p* values for the most significant ontology term for each cluster.

Epithelial cell clusters (cluster 9, 14, 15, 16, 17, 19, 23) were further divided according to the expression levels of keratin isoforms and their DEGs (Figures 2A–C and Supplementary Figure S2). They were largely discriminated by the expression of *KRT7* and *KRT19* in simple ductal epithelia and of *KRT5* and *KRT14* in stratified epithelia [13] (Figure 2A). In detail, the cluster 19 with elevated expression of secretory genes *MUCL1* and *PIP*, lipid metabolism genes *GLYATL2*, *SERHL2*, and *UGT2B28* and holocrine secretion gene *MGST1* [14] was consistent with lipid-producing sebaceous gland cells (Figures 2B,C and Supplementary Figure S2). Similarly, the clusters 17, 23, 16, 14 and 15 were defined sweat gland cells, lung secretory cells, mucin-producing ductal cells, mixed/epidermal differentiating keratinocyte and basal keratinocytes, respectively, indicating multi-organ nature of OMCT epithelial cell compositions (Figures 2B,C and Supplementary Figure S2). The GO enrichment analysis of the gene signatures also supported the association of markers with relevant cell types and processes (Figure 2D). Expression status of the epithelial cell clusters were well matched with those of reference datasets [15–17] for normal cell types (Supplementary Figure S3A). When compared to the Skin Signatures database [18], transcription signatures of the clusters in OMCTs were well matched with specific skin cells (Supplementary Figure S3B). Six fibroblasts/stromal cell clusters were largely categorized into fibroblasts (clusters 0, 4, 26) and ovarian stromal cells (clusters 1, 7, 8) based on their respective expression of *COL1A1*/*COL1A2* and steroidogenesis-related genes *STAR* and *FHL2* (Figure 2E). The cluster 26 expressed dermal papillary fibroblast-related genes *PTGDS* and *MGP* (Figure 2F and Supplementary Figure S4). The clusters 0 and 4 expressed chemokines *CXCL12* and *CCL2*, and complement genes *C3* and *CFD*, consistent with pro-inflammatory fibroblasts (Figure 2F and Supplementary Figure S4). The clusters 1, 7 and 8 highly expressed steroidogenic genes *STAR* as well as tissue remodeling genes *HAS1*, *ADAMTS1* and *ADAMTS4,* consistent with theca-stromal cells from ovary [9] (Figure 2F and Supplementary Figure S4), which might be included from non-tumor tissues from ovaries. The fibroblast clusters were enriched for collagen containing extracellular matrix, whereas the stromal cell clusters were enriched for response to lipid, endogenous stimulus or hormone in GO analysis (Figure 2G).

## Discussion

The OMCT is a distinct tumor in which heterogenous tissues mimicking mature multi-organ tissues with predominant skin components [1, 2]. The aim of this study was to address transcription profiles of heterogenous cell types of OMCTs, which had not been identified. For this, we adopted the scRNA-seq and found that transcriptomes of individual cell types in OMCTs were not different from those of normal cells in orthotopic locations. Although the OMCT arises from an ovum without a sperm (parthenogenic), our data indicate that the uniparental chromosomes may not affect the expression of individual tissues in OMCTs. The parthenogenesis might lead the ovum to an OMCT instead of leading to embryo development. The limitation of our study was to analyze only two cases. Although OMCT is a benign tumor with relatively uniform clinical features, analysis of a larger cohort will be needed to solidify our results.

The scRNA-seq is a high-resolution assay used to interrogate transcriptome of individual cells within tissues that may help find novel discoveries in tissue heterogeneity [9, 19, 20]. For example, in cancer, scRNA-seq of individual cells may give insight into the existence and behavior of different cell types in both tumor and microenvironment cells [19, 20]. The scRNA-seq is becoming widely used across biological field including developmental biology, oncology, immunology and Infectious diseases [20]. In onclogy, scRNA-seq analyzed diverse cancer types including cancers of breast, colon and ovary. The scRNA-seq for serous ovarian carcinomas identified heterogenous expression profiles of cancer cells as well as ascitic inflammatory cells that might alter disease progression and treatment responses [19]. In this study, we for the first time disclosed the transcriptome profiles of OMCTs at a single cell level and found that they may recapitulate expressions of normal counterparts. Our study could be an example to analyze other gynecologic diseases with heterogenous tissues such as other germ cell tumors using scRNA-seq.

## Data Availability

The datasets presented in this study can be found in online repositories. The names of the repository/repositories and accession number(s) can be found in the article/Supplementary Material.
