# Supplementary material for: Single Cell Gene Transcriptome Analysis of Ovarian Mature Teratomas
Source: Pathol Oncol Res. 2021 Apr 16;27:604228. doi: 10.3389/pore.2021.604228 (PMC8262198; doi:10.3389/pore.2021.604228)
Supplement: Supplementary file 1 [file DataSheet1.pdf]

Single cell gene expression in ovarian mature teratomas

<Supplementary information>

1. Figures

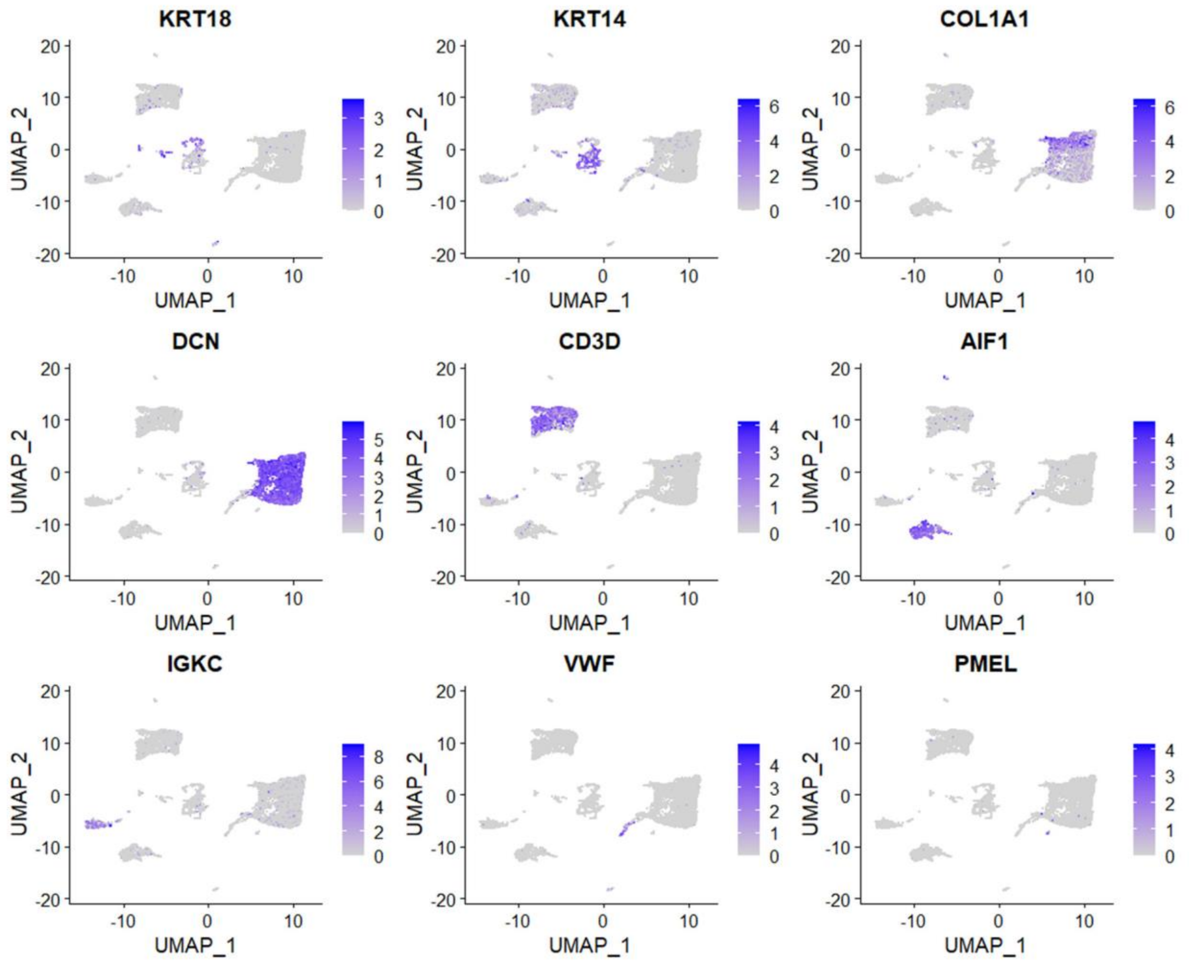

Supplementary Fig. 1. UMAP plot showing cell-specific markers. Relative expression of cell-specific markers for epithelial cells/keratinocytes (*KRT18*, *KRT14*), stromal cells/fibroblasts (*COL1A1*, *DCN*), T cells (*CD3D*), macrophages (*AIF1*), B cells (*IGKC*), endothelial cells (*VWF*) and melanocytes (*PMEL*).

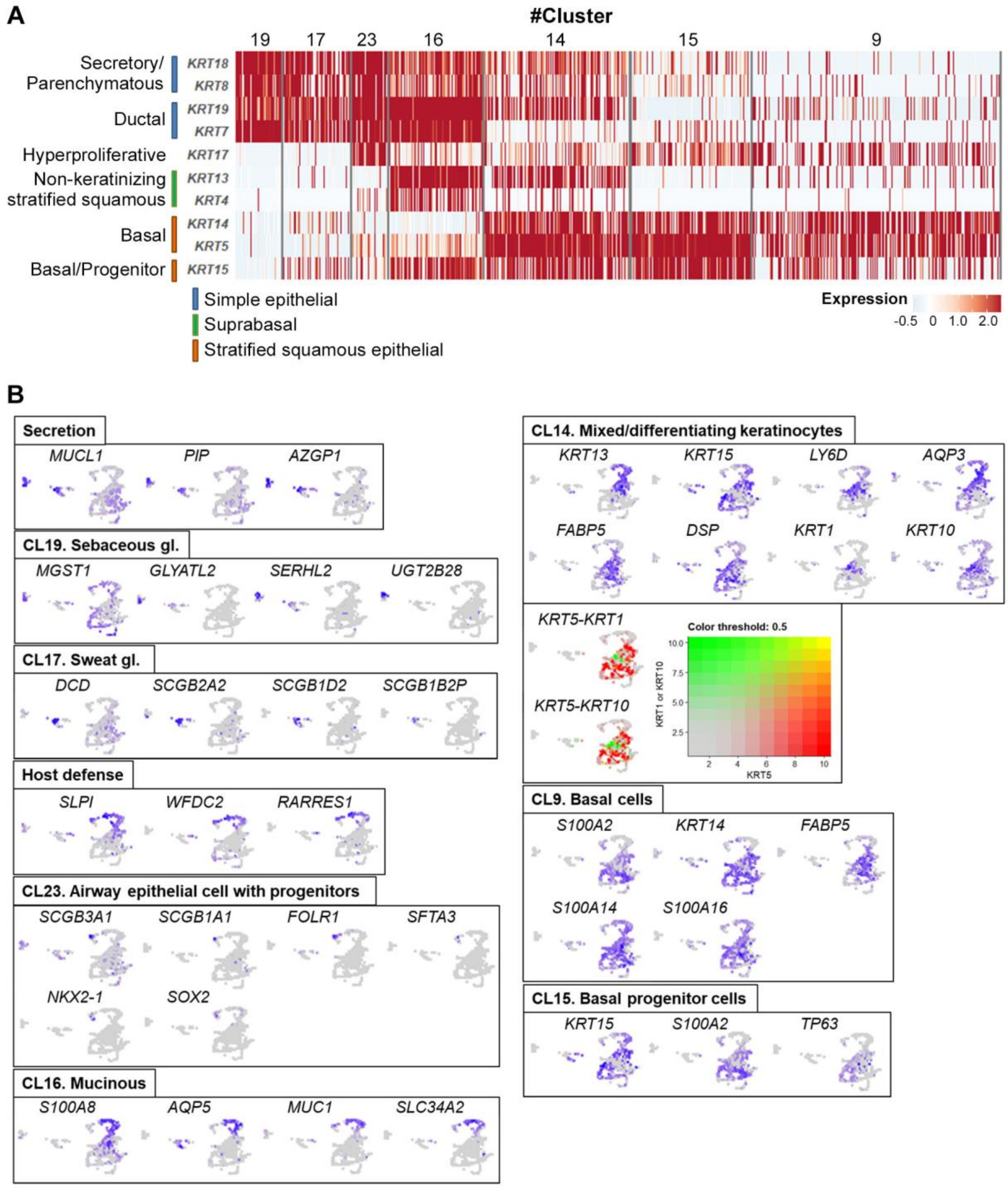

Supplementary Fig. 2. Keratin expression and differentially expressed genes of epithelial cell clusters. (A) Heatmap of keratin isoforms for epithelial cell clusters. The left bar indicates epithelial type. (B) Differentially expressed genes (DEGs) of epithelial cell clusters. Relative expression of each marker was shown from lowest expression (gray dots) to highest expression (purple dots). Cluster 14 expressed suprabasal marker *KRT1*/*KRT10* and basal marker *KRT5*/*KRT14*, but *KRT1*+ or *KRT10*+ cells in cluster 14 did not express *KRT5*.

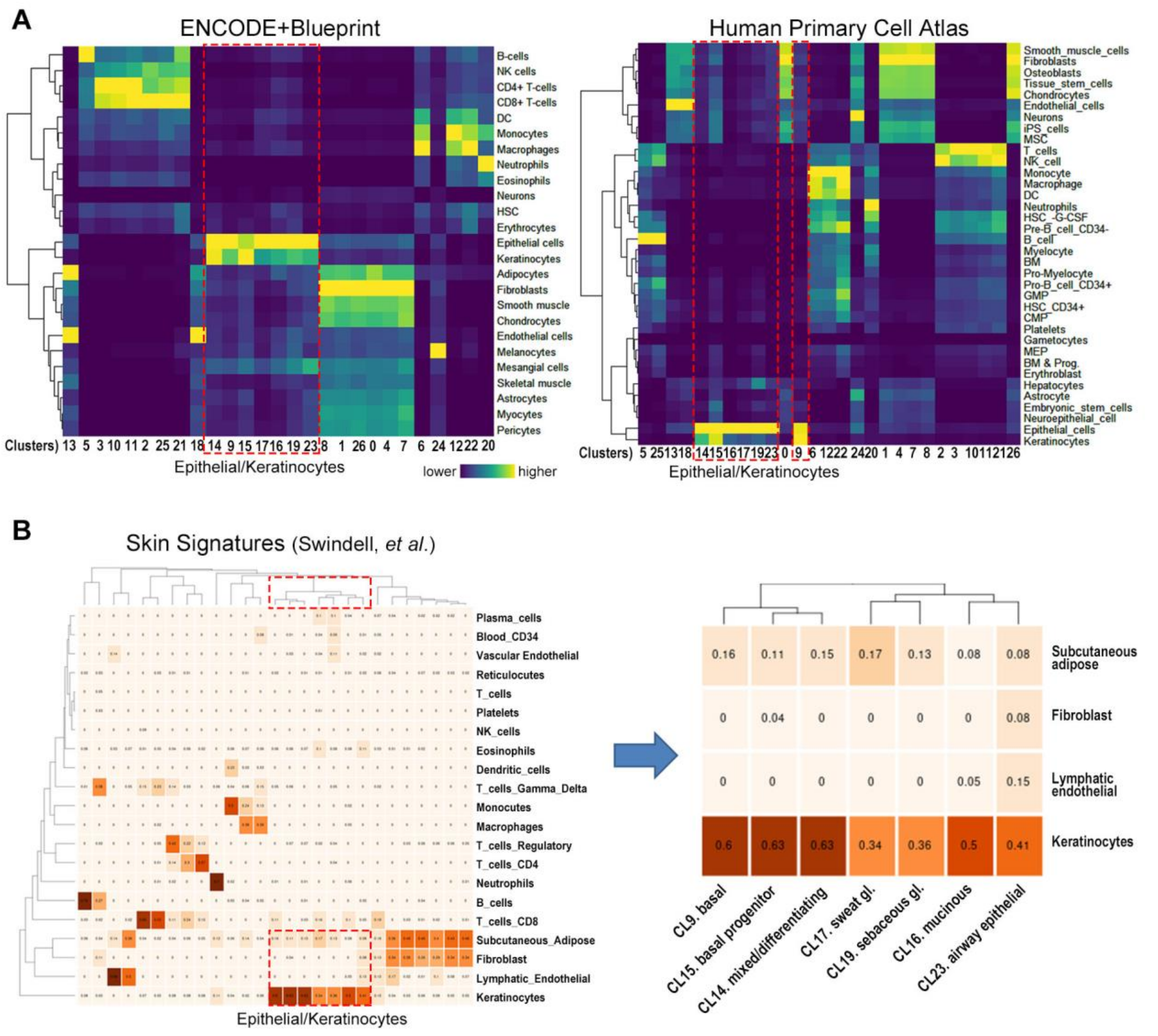

Supplementary Fig. 3. Reference-based cluster annotation. (A) Heatmap of SingleR annotation scores derived by reference to the ENCODE + Blueprint and Human Primary Cell Atlas database with clusters. (B) Predicted cell types of clusters derived by reference to the Skin Signatures.

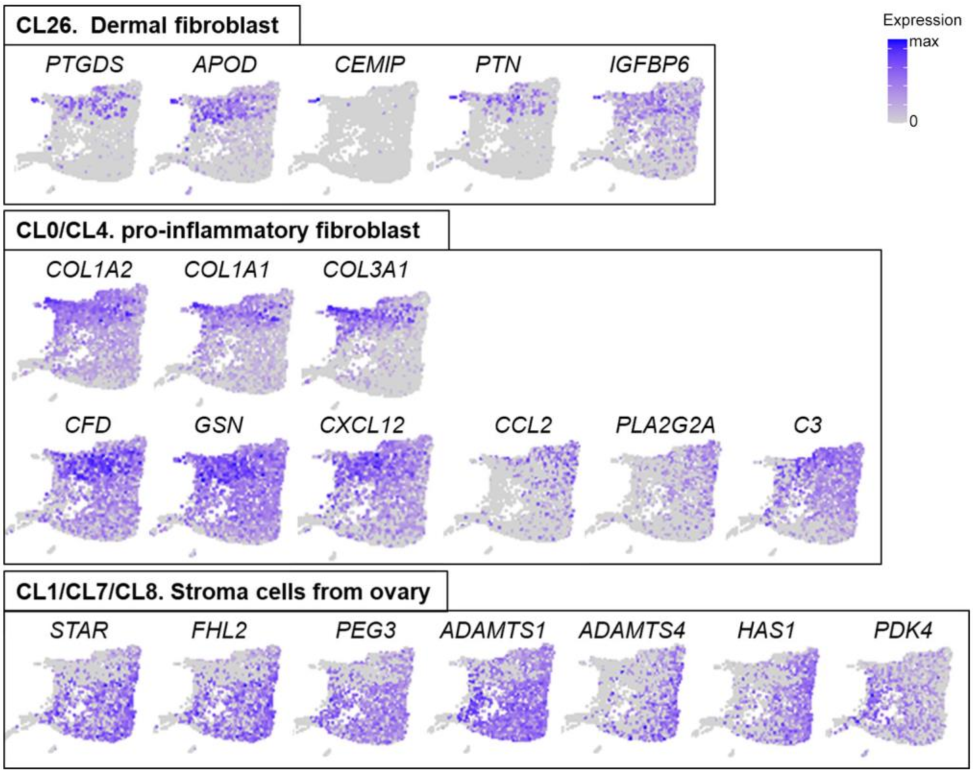

Supplementary Fig. 4. Differentially expressed genes of fibroblast/stromal cell clusters. Relative expression of each marker was shown from lowest expression (gray dots) to highest expression (purple dots).
